# Bone marrow mesenchymal stromal cells in a 3D system produce higher concentration of extracellular vesicles (EVs) with increased complexity and enhanced neuronal growth properties

**DOI:** 10.1186/s13287-022-03128-z

**Published:** 2022-08-19

**Authors:** Elmira Jalilian, Hamed Massoumi, Bianca Bigit, Sohil Amin, Eitan A. Katz, Victor H. Guaiquil, Khandaker N. Anwar, Peiman Hematti, Mark I. Rosenblatt, Ali R. Djalilian

**Affiliations:** 1 Department of Ophthalmology and Visual Sciences, Illinois Eye and Ear Infirmary, University of Illinois at Chicago, 1855 W. Taylor Street, MC 648, Chicago, IL 60612 USA; 2 Richard and Loan Hill Department of Bioengineering, University of Illinois at Chicago, Chicago, IL USA; 3 Department of Medicine, Hematology/Oncology Division, University of Wisconsin-Madison, School of Medicine and Public Health, Madison, WI 53705 USA

**Keywords:** Extracellular vesicles, Exosomes, Bone marrow mesenchymal stromal cell, 3D culture, 2D culture, Neuronal growth

## Abstract

**Purpose:**

Extracellular vesicles (EVs) derived from mesenchymal stromal cells (MSCs) have been demonstrated to possess great potential in preclinical models. An efficient biomanufacturing platform is necessary for scale up production for clinical therapeutic applications. The aim of this study is to investigate the potential differences in neuro-regenerative properties of MSC-derived EVs generated in 2D versus 3D culture systems.

**Method:**

Human bone marrow MSCs (BM-MSCs) were cultured in 2D monolayer and 3D bioreactor systems. EVs were isolated using ultracentrifugation followed by size and concentration measurements utilizing dynamic light scattering (NanoSight) and by fluorescence staining (ExoView). Mouse trigeminal ganglia (TG) neurons were isolated from BALB/c mice and cultured in the presence or absence of EVs derived from 2D or 3D culture systems. Neuronal growth and morphology were monitored over 5 days followed by immunostaining for β3 tubulin. Confocal images were analyzed by Neurolucida software to obtain the density and length of the neurites.

**Results:**

The NanoSight tracking analysis revealed a remarkable increase (24-fold change) in the concentration of EVs obtained from the 3D versus 2D culture condition. ExoView analysis showed a significantly higher concentration of CD63, CD81, and CD9 markers in the EVs derived from 3D versus 2D conditions. Furthermore, a notable shift toward a more heterogeneous phenotype was observed in the 3D-derived EVs compared to those from 2D culture systems. EVs derived from both culture conditions remarkably induced neurite growth and elongation after 5 days in culture compared to untreated control. Neurolucida analysis of the immunostaining images (β3 tubulin) showed a significant increase in neurite length in TG neurons treated with 3D- versus 2D-derived EVs (3301.5 μm vs. 1860.5 μm, *P* < 0.05). Finally, Sholl analysis demonstrated a significant increase in complexity of the neuronal growth in neurons treated with 3D- versus 2D-derived EVs (*P* < 0.05).

**Conclusion:**

This study highlights considerable differences in EVs obtained from different culture microenvironments, which could have implications for their therapeutic effects and potency. The 3D culture system seems to provide a preferred environment that modulates the paracrine function of the cells and the release of a higher number of EVs with enhanced biophysical properties and functions in the context of neurite elongation and growth.

**Supplementary Information:**

The online version contains supplementary material available at 10.1186/s13287-022-03128-z.

## Highlights

- Extracellular vesicles (EV) derived from 2D and 3D bone marrow mesenchymal stromal cells (BM-MSCs) demonstrate different characterization phenotype.
- Heterogeneity and concentration of BM-MSCs-derived extracellular vesicles are highly impacted by the culture conditions.
- 3D-derived EVs can significantly enhance trigeminal nerves elongation and complexity compared to 2D-derived EVs.
- 3D culture conditions are highly efficient environment for upscale production of MSC-derived EVs with enhanced neuro-regeneration properties for corneal nerve regeneration.

**Supplementary Information:**

The online version contains supplementary material available at 10.1186/s13287-022-03128-z.

## Introduction

The cornea is the most densely innervated tissue in the human body [1]. Corneal nerves play a critical role in maintaining ocular surface health and homeostasis [2–4]. Disruption of corneal nerves often leads to serious detrimental effects [5, 6] that can lead to lost or compromised innervation and result in neurotrophic keratopathy and blindness [2, 7–9]. Following injury, corneal nerves may regenerate over many years; however, sub-basal nerve density never returns to normal [10, 11]. Current treatment options such as eye drops and surgeries are not very effective [10, 12] and often fail to manage symptoms in moderate to severe cases [13]. Recent studies have shown the beneficial effects of mesenchymal stem cell-based (MSCs) and their secreted factors as a therapeutic approach for different ocular diseases.

MSCs primarily exert their therapeutic effects mainly through their paracrine factors, particularly a type of extracellular vesicles (EVs) called exosomes [14–17]. MSCs primarily exert their therapeutic effects mainly through their paracrine factors, particularly a type of extracellular vesicles (EVs) called exosomes. Exosomes are a subtype of EV formed by an endosomal route and are typically 50–150 nm in diameter that carrying out the biological functions of MSCs for tissue repair and regeneration [18, 19]. Exosomes offer a considerable advantage over cells. While the use of MSCs has potential side effects (e.g., immunogenicity and tumorigenicity) along with poor engraftment, exosomes do not present this risk and can be easily transported and stored with long-term stability [20, 21]. Based on that premise, manufacturing EV products to be used as a third party “off the shelf” can be readily scalable at a lower cost compared to cell therapy approaches, thus, merited attention among scientific communities [22–27].

The beneficial effects exerted by MSC-exosomes have been highlighted in different Central Nervous System (CNS) and peripheral nerve injuries animal studies [2, 28–33]. In respect to ocular diseases, the therapeutic effects of EVs including their wound healing capacity and angiogenesis effects have been under the spotlight recently [34–36]. We previously showed that a human corneal MSC (cMSC) secretome is able to inhibit corneal neovascularization [32], and modulate macrophages toward an anti-angiogenic and anti-inflammatory immunophenotype [33], suggesting that human cMSC secreted factors may be used therapeutically in ocular surface diseases. In addition, local administration of umbilical cord MSC-exosomes on mice with autoimmune uveitis demonstrated the reduced intensity of autoimmune uveitis in response to reduced infiltration of T cells [37].

It has been reported that EVs deliver their therapeutic functions through their associated cargo that is comprised of proteins and nucleic acids (e.g., DNA, mRNA, and miRNA.) [38] The protein composition of EVs, including tetraspanins, peripheral membrane proteins, and cytosolic proteins, has been shown to be associated with substantial functional changes [39]. EVs carrying the argonaute-2 (AGO-2) protein promote significant axonal regeneration and survival of ganglion cells from the retina [40]. Among the miRNAs most frequently associated with MSC-EVs' therapeutic properties, miR-21, miR-17-92, and miR-133b are linked to neural damage [41].

Furthermore, EVs isolated from different cell culture conditions have shown to have a different heterogeneous mixture of subpopulations with specific protein profiles [42–44]. So far, many 2D cell culture systems have been widely employed as the “gold standard” for EV generation [45]. However, in a 3D culture system, growth behavior, cell morphology, and cell-to-cell interactions within the extracellular matrix are very different compared to a 2D culture system [46]. Studies have shown that EVs derived from 3D culture have altered EV secretion dynamics and molecular contents compared to the 2D culture-derived EVs [47]. Studies have shown that EVs derived from 3D culture have altered EV secretion dynamics and molecular contents compared to the 2D-derived EV. Thippabhotla et al. demonstrated a highly similar RNA profile (~ 96%) among EVs obtained from a 3D culture system and in vivo circulating EVs. Whereas EVs obtained from 2D culture systems had a closer correlation with their parent cells' RNA profile. Zhang et al. demonstrated that the difference in the content of exosomes derived from a 3D culture is responsible for their distinctive higher therapeutic efficacy on traumatic brain injury [48]. Moreover, in a comparative study between 3D- versus 2D-obtained exosomes, Villasante et al. conveyed that the 3D model closely replicated patient's plasma exosomes in carrying high levels of the polycomb histone methyltransferase EZH2 mRNA compared to the 2D-obtained exosomes [49].

However, the therapeutic effects of EVs generated in different cell culture environments have not been systematically studied with respect to their neuro-regenerative properties in sensory neurons such as those that innervate the cornea. Therefore, the main goal of the present study was to produce BM-MSCs-derived EVs from two different cell culture conditions and study their regenerative effect on neuronal growth and elongation.

## Materials and methods

### Cell culture expansion

Bone marrow MSCs used in this study were obtained from three human donors previously isolated in Dr. Peiman Hematti’s laboratory [50] and grown at 37 °C in 5% CO_2_. Cells were incubated and grown in Nunclon Delta Surface Treated T75 cm^2^ flasks (Thermo Scientific, #156499) to 80% confluency with hMSC expansion media (High-Performance Media Kit (RoosterBio, #KT-016) supplemented with 1% Antibiotic, Antimycotic Solution (Corning, #30-004-Cl)). Once confluent, cells were washed once with 1 × Phosphate Buffered Saline (PBS) (Sigma, #806552) and incubated for 5 min with TrypLE™Express (TrypLE) detachment solution (Gibco, #12604-021) and neutralized with fresh media once detached. Cells were spun at 280×*g* for 5 min, resuspended in fresh media, and counted by hemocytometer for 2D and 3D experiments. All cells were grown from passages 3–5 for this experiment.

### 2D culture

Cells were seeded at a density of 13,000 cells/cm^2^ in T75 cm^2^ flasks and left in culture for 72 h for monolayer confluency of about 80%. Then, cells were washed twice with 1 × PBS and incubated with a collection medium (RoosterCollect™-EV, M2001) for an additional 72 h. After the incubation period, media was collected and ultracentrifuged to isolate the secreted EVs, and cells were then counted to determine cell growth rates from initial seeding density.

### 3D culture

In parallel to 2D culture, BM-MSCs were seeded with 1.25 g of Low Concentration Synthemax II Microcarriers (Corning, #3781) in a Vertical-Wheel™ Bioreactor (PBS Biotech, #0.1 MAG IA-0.1-D-001) at a density of 4600 cells/cm^2^, as per manufacturer’s instructions. Microcarriers were initially incubated with 20 mL of expansion media in the bioreactor to equilibrate while cells were counted. Once prepared, cells were added to the bioreactor and kept undisturbed for 20 min without rotation, swirled once, and left for an additional 10 min unagitated leaving the cells to adhere to the microcarriers. After cell adhesion, the final expansion media level was brought up to 90 mL and the bioreactor wheel was programmed to rotate at 25 rpm. Cells were left to grow for 72 h before receiving a 2 mL feed of media and a rotation speed increase to 35 rpm. On day 5 of culture, the bioreactor was washed twice with 1 × PBS, and collection medium (RoosterCollect™-EV, M2001) was added to the culture for an additional 72 h. After the starvation period, the wheel was stopped and microcarriers were left to settle down at the bottom of the bioreactor. Thereafter, the collection media was carefully collected. In order to count the adhered cells on the microcarriers, beads were washed twice with 1 × PBS and incubated at 37 °C with 50 mL TrypLE detachment solution for 15 min without agitation, and then, an additional 20 min at 40 rpm. Once detached, cells and microcarriers were separated and the 50 mL solution was used to obtain the final cell count.

### EV isolation

Following the collection of collection media from 2 and 3D culture experiments, EVs were isolated by ultracentrifugation. For this, collection media was passed through a 100 µm nylon cell strainer (Corning, #352360) and centrifuged for 15 min at 2000×*g* at 4 °C. The supernatant was transferred to Open-Top Thinwall Ultra-Clear Tubes (Beckman Coulter, #C13926) in 3.5 mL aliquots and the tubes were ultracentrifuged for 4 h at 4 °C, at a speed of 27,000 rpm. Then, the supernatant was collected and used as “depleted media,” while the remaining 200 µL of the inoculum was left undisturbed for resuspending and collecting the “EVs.” All samples were stored at − 80 °C for further analyses.

### Analysis of EV size and quantification

To characterize EVs’ size and concentration, the NanoSight NS300 (Malvern Panalytical, Worcestershire, UK) was used, which utilizes Nanoparticle Tracking Analysis (NTA) with NTA 3.1 software (NanoSight, UK). The EV suspension was diluted in double-filtered PBS (1:50) to obtain approximately 40–100 particles per field. Then, 1 mL of EVs suspension in PBS was transferred to a syringe, the air bubbles were carefully removed, and the syringe was inserted into an O-ring top plate NTA chamber. Particle scattering of 405 nm light was recorded by a CCD camera (three videos of 30 s each, camera level = 14, detection threshold = 3), and the Brownian motion was determined frame-by-frame with three scattering measurements for size and density recorded per sample.

### EV analyses with ExoView (NanoView Biosciences, USA)

The exosome markers were analyzed using the single particle interferometric reflectance imaging sensor with the ExoView platform (NanoView Biosciences, USA). Briefly, the ExoView tetraspanin kit with immobilized antibodies against the tetraspanins CD9, CD63, and CD81 on silicon dioxide chips (NanoView Biosciences, Boston, MA) was used to capture 2D and 3D-derived EVs. Briefly, samples were diluted in PBS with 0.5% Tween-20 (PBST) and then incubated on ExoView Tetraspanin Chip for 16 h at room temperature in a 24-well plate. Chips were then washed three times in 1 ml PBST for 3 min on an orbital shaker. The chips were then incubated for 1 h at RT in a cocktail of fluorescent antibodies comprised of anti-CD9-AF647; anti-CD63-AF488; anti-CD81-AF555 diluted in 5% bovine serum albumin. After careful rinsing and drying steps, image acquisition from each chip was carried out using the ExoView® R100 platform, and the data were analyzed by the NanoViewer 2.9 along with ExoViewer 3 (NanoView Biosciences), respectively.

### Trigeminal ganglia (TG) neuronal growth assay

All animal experiments were conducted in compliance with the approved animal protocols by the Animal Care and Use Committee at the University of Illinois at Chicago and according to the guidelines of the Association for Research in Vision and Ophthalmology Statement for the Use of Animals in Ophthalmic and Vision Research and in compliance with the Arrive guidelines. Sensory neurons from mice TG were isolated and cultured as previously described [51]. Briefly, 3–5-week-old BALB/c mice were euthanized, and TG pairs were removed and digested in a 60 U papain solution (Worthington) at 37 °C for 18 min. The papain solution was decanted, and TGs were transferred into a mixture of type II collagenase (Worthington, Lakewood, NJ) and type II dispase (Thermo Fisher) for another 18 min at 37 °C. Finally, the isolated cells were separated from myelin and debris into a single-cell suspension using a Percoll gradient before being cultured on 35 mm dishes with a 20 mm glass bottom well (Cellvis, Mountain View, CA). Poly-d-lysine (PDL) was used to coat the glass bottom of the dish. Neuronal cells were cultured in DMEM/F12 medium and replaced with collection media plus treatments (EVs or depleted media) on day 2 when neuronal cells exhibited minimum neurite growth. Two doses of BM-MSC-derived EVs (4 × 10^10^ particles or 40 × 10^11^ particles/dish) were used as treatments, while control dishes remained intact in RoosterCollect™-EV solution. Cell viability and neurite growth were monitored under a bright-field microscope for 4 days post-treatment. The experimental design was repeated four times.

### Beta 3 tubulin immunofluorescent staining of TG neurons

Following the EV treatment, on day 5 of culture, TG neurons were washed once with 1 × PBS and fixed in 4% paraformaldehyde (PFA, Ted Pella, #18505) for 15 min. Samples were washed three times with 1 × PBS for 5 min each with gentle agitation. To permeabilize the cell membrane, samples were incubated in 1 × PBS + 0.1% Triton-X-100 (Fisher, #BP151-100) for 1 h at room temperature (RT). Next, samples were incubated in a blocking buffer containing 1 × PBS, 0.05% Tween-20, and 1% BSA for 1 h at RT and then incubated in the dark with a FITC-conjugated antibody against B3 tubulin (Biolegend, #801203) at a concentration of 1:300 dilution for 90 min at RT. Cells were then washed with PBST (PBS + 0.05% Tween-20) three times for 10 min each with gentle agitation. Following washes, samples were incubated with nuclear stain Hoechst 33,342 (in a 1:5000 dilution) for 5 min. After incubation, samples were washed three times with 1 × PBS for 10 min and stored in 1 × PBS for confocal imaging (Zeiss, LSM 800).

### TG neurite length analysis

The stained neurons were imaged using a confocal microscope (Z1; Carl Zeiss Meditec, Jena, Germany). Five randomized squares of 2 × 2 mm regions of interest were chosen per dish, and the neurons were imaged using a 20X magnification lens. Neuronal growth and complexity analyses were performed using Neurolucida tracing software (MBF Biosciences). Total length tracing and the Sholl analyses were utilized to represent the neuronal growth and complexity of each cell, respectively.

### Statistical analysis

Prism version 8.4.2 (GraphPad) was used for all statistical analyses. For all the other studies, the significant difference between test groups was evaluated using a two-tailed, unpaired Student’s t test. A *P* value < 0.05 was considered statistically different.

## Results

### MSC grown in 3D conditions produced significantly higher cell number

**Fig. 1:**
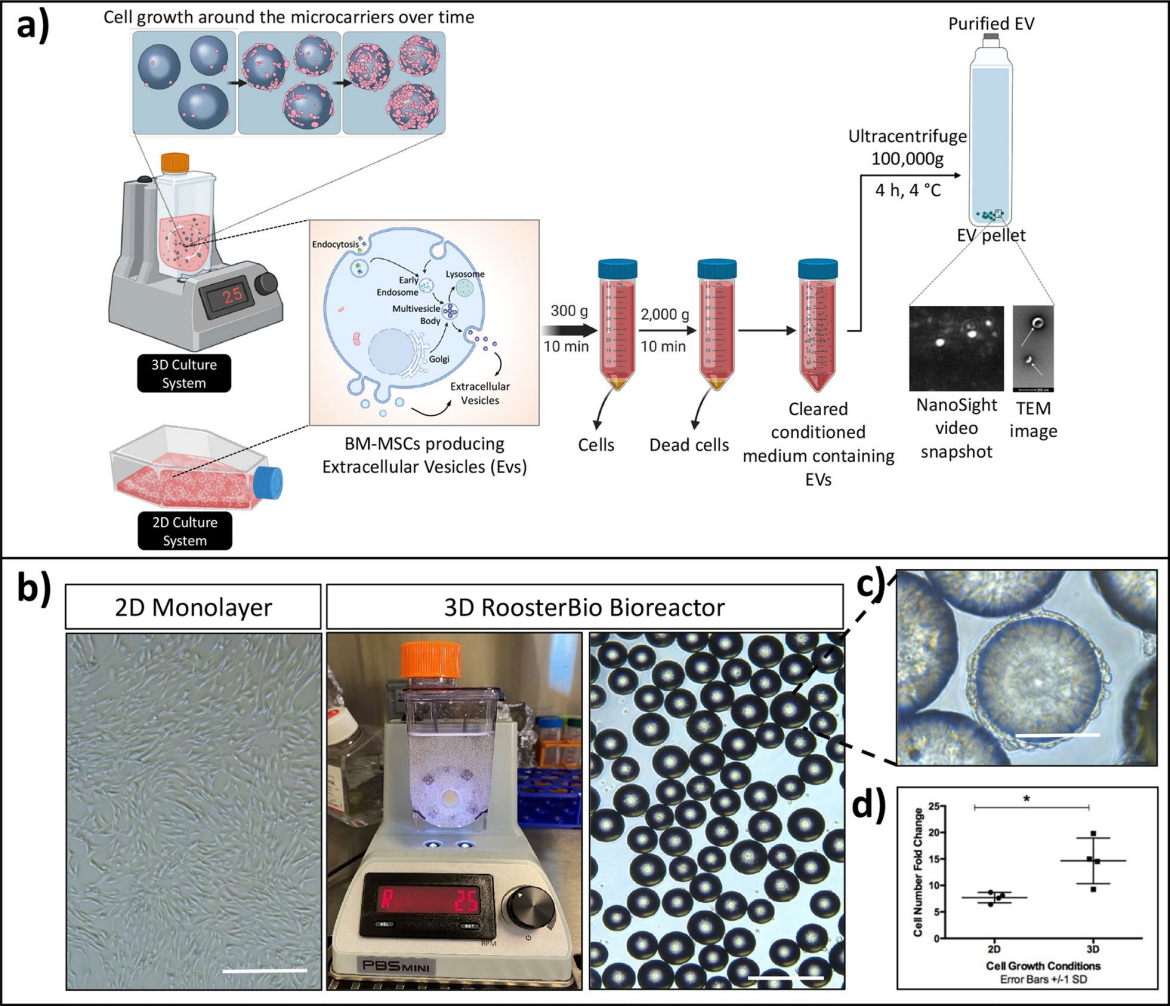
2D versus 3D culture system for BM-MSCs. **a** Schematic diagram of BM-MSCs grown in a 2D and 3D culture conditions. Cells were either grown in 2D culture flask or 3D bioreactor on microcarriers for expansion. Cells were transferred to collection medium for 3 days and then collected and processed for EV isolation using low and high-speed centrifugation steps. **b** Morphology of BM-MSCs after 3 days in collection medium and **c** bioreactor, microcarriers and cell attachment and expansion around microcarriers. **d** The statistical analysis illustrated significant expansion in cell number in 3D versus 2D culture conditions (*P* < 0.05). Scale bar 200 px

BM-MSCs (2 × 10^6^) at passage 3 were either plated in a 2D flask culture or a 3D Vertical-Wheel™ bioreactor using Low Concentration Synthemax II Microcarriers as per recommended by the manufacturer (Fig. 1). The schematic diagram of BM-MSCs expansion and extracellular vesicles (EVs) collection from 2 and 3D culture is shown in (Fig. 1a). When cells reached around 80% confluency, (Fig. 1b, c) the media were switched to a chemically defined basal media (collection medium), and the supernatant was collected after 72 h and processed for EV isolation (details are described in methods). Fold-change in cell number is illustrated with an average of 6.9 ± 3.7 and 3.8 ± 0.8 for 3D and 2D cell cultures, respectively, which was statistically significant (Fig. 1d, *P* < 0.005). The EV-depleted culture media were preserved (w/o-EVs) after EV isolation from collection media by ultracentrifugation and used as the control treatment.

### MSC grown on 3D culture generated higher EVs content

**Fig. 2:**
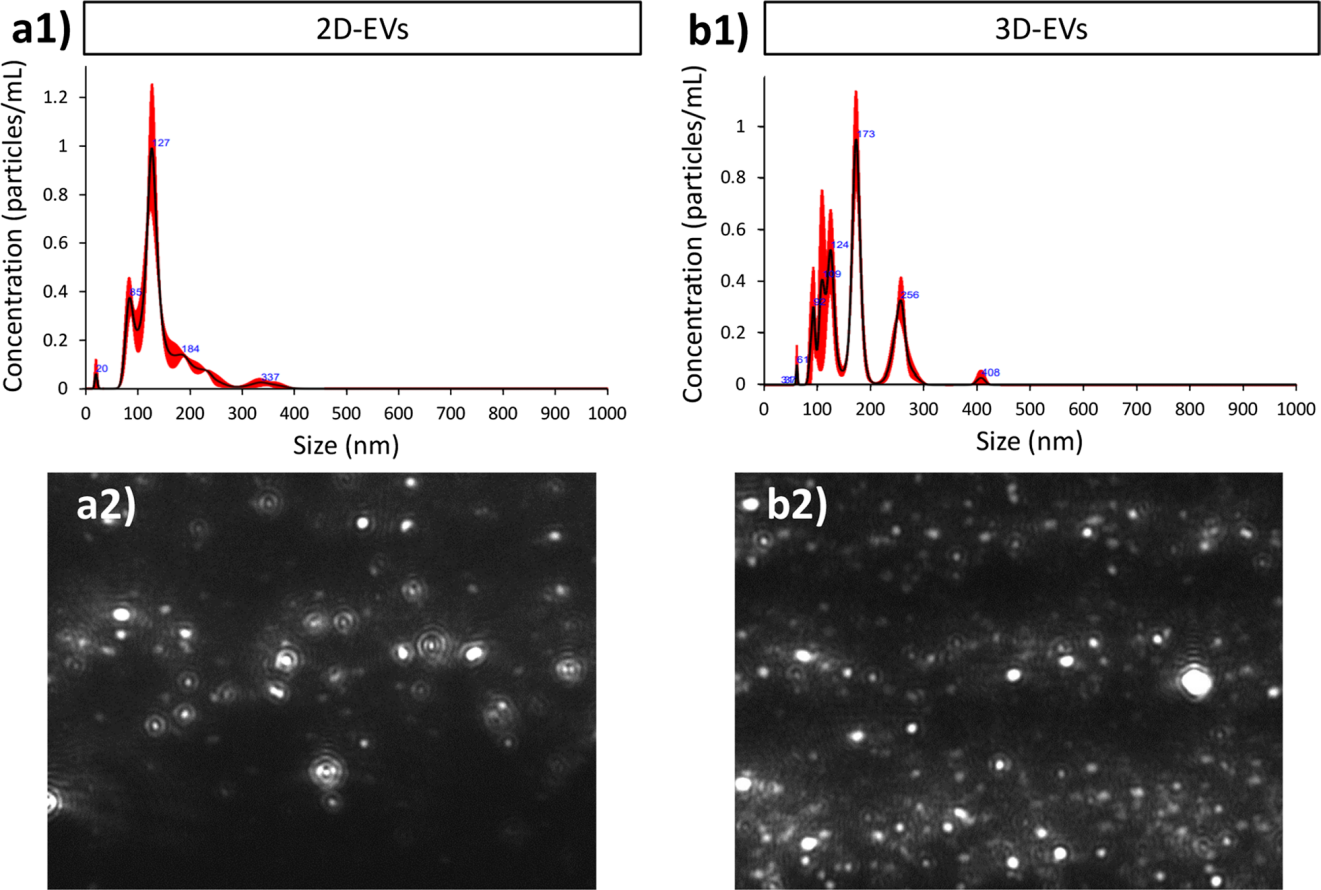
Characterization of particles from 2 and 3D EVs using NTA analysis. Size distribution of particles using NTA in **a1** 2D and **b1** 3D condition. Representative image frame of EVs extracted form a **a2** 2D culture system and a **b2** 3D culture system

We performed quantitative and qualitative comparisons of the isolated EVs from both 2D and 3D culture systems. The size distribution and concentration of EVs evaluated by the Nanoparticle Tracking Analysis (NTA) showed that most of the EVs’ sizes are in the range of 60–180 nm in 2D samples, but a less abundant population of EVs was also detected with larger sizes between 300 and 400 nm (Fig. 2a1, a2). Regarding the 3D culture system, NTA analysis illustrated three size distributions. Most of the EVs were detected between 50 and 170 nm. The second population with less abundance was detected between 200 and 300 nm, and a very small peak was observed around 400 nm (Fig. 2b1, b2). The mean diameters shown by NTA analysis for 2D and 3D-derived EVs were 139.13 nm and 146.86 nm, respectively. However, further analysis showed a 28-fold increase in the average concentration of EVs/mL in the 3D versus 2D culture system. Although there was a trend toward an increase in the number of EVs, this difference was not statistically significant (Additional file 1: Figure S1). Videos demonstrating an example of the dynamic light scattering method for each of the 2D-EV and 3D-EV samples can be found in the Additional file 1: Figure S1.

### MSC grown on 3D culture produced more heterogeneous exosomes

**Fig. 3:**
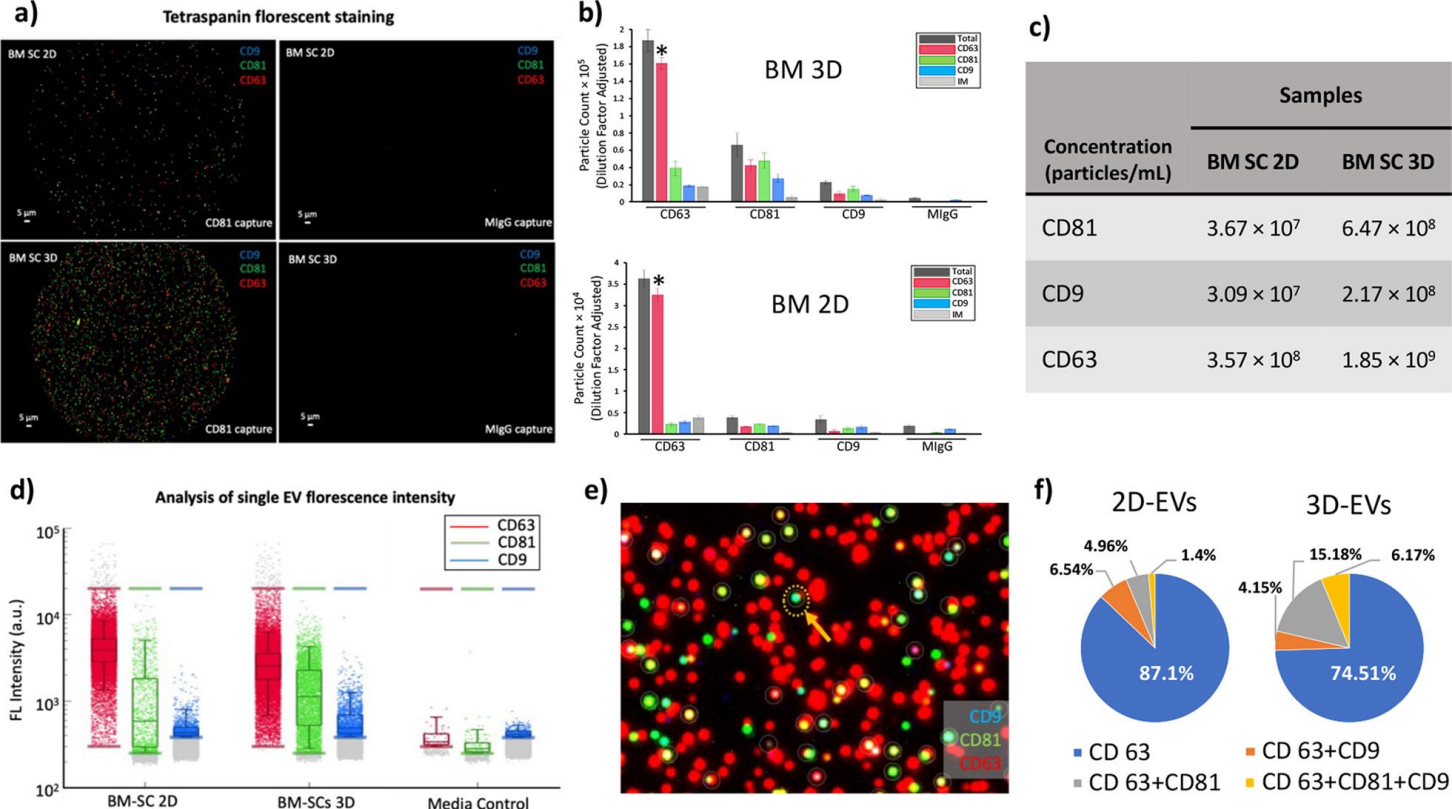
Characterization of 2D and 3D EVs using ExoView. **a** Tetraspanin antibodies-capture allows for the detection and identification of subpopulations according to the different tetraspanin compounds expressed on each particle (right positive stained, left IgG control). **b** Copy number of each type of particle from 2 and 3D cell culture counted and presented in a bar-graph with a tenfold increase on the Y-axis in BM 3D graph versus that of BM 2D (*P* < 0.05). **c** Concentration of each marker per milliliter of the extraction medium. **d** Fluorescence intensity per EV was measured for each channel. **e** Representative image of capture spot of colocalization. **f** Representative pie charts from a single chip that exhibits the percentage of total particles detected with either CD63 (blue), CD63 + CD9 (orange), CD 63 + CD81 (gray), or CD 63 + CD81 + CD9 (yellow) on different capture spots

The expression of exosome markers was performed using the ExoView platform (NanoView Biosciences, USA). Staining with fluorescent tetraspanin antibodies allowed quantification of all tetraspanin-positive particles, regardless of size (Fig. 3a). All particles expressing a copy of one of these markers were counted and are shown in Fig. 3b. The concentrations of CD81, CD9, and CD63 positive EVs per milliliter are also shown in Fig. 3c. Both 2D and 3D samples predominately contained CD63^+^ EVs. However, there was a notable shift toward a more heterogeneous phenotype in the 3D condition, with a remarkable increase in the concentration of CD81 and CD9 positive vesicles. Overall particle concentration also increased in 3D culture conditions. Additionally, fluorescence intensity per EV was measured for each channel. A significant increase was observed in the mean fluorescence intensity of CD81 and CD9 positive particles in 3D samples compared to their 2D counterparts (Fig. 3e). This indicates that in addition to an increase in the number of CD81 and CD9 positive vesicles in the 3D samples, each vesicle is binding a higher number of fluorescent antibodies, which is an indication of an increase in the number of CD81 and CD9 epitopes per EV.

Fluorescence data were exploited to analyze the degree of colocalization of different markers. The software analyzes the number of different fluorophores present on each vesicle, allowing detailed analysis of vesicle subpopulations. A representative image of the captured spot of colocalization is shown in Fig. 3d, and triple positive (CD9/CD81/CD63) EVs are circled. For example, analysis of CD63 captured EVs showed a shift in tetraspanin phenotype in the 3D samples compared to the 2D. Data illustrated that the percentage of CD63 captured EVs positive for all three tetraspanins increased from 1.4% to 6.2% in 3D culture. This could confirm the increased heterogeneity of EVs from cells cultured in 3D conditions (Fig. 3f).

The average colocalization percentage is shown in Additional file 1: Figure S2. Furthermore, the size of each marker (particles between 50 and 200 nm) was measured by interferometry-based label-free measurements and the data showed no significant difference in the size of the particles within the three markers (Additional file 1: Figure S3).

### Human BM-MSC-Derived EVs induced neurite elongation over time

**Fig. 4:**
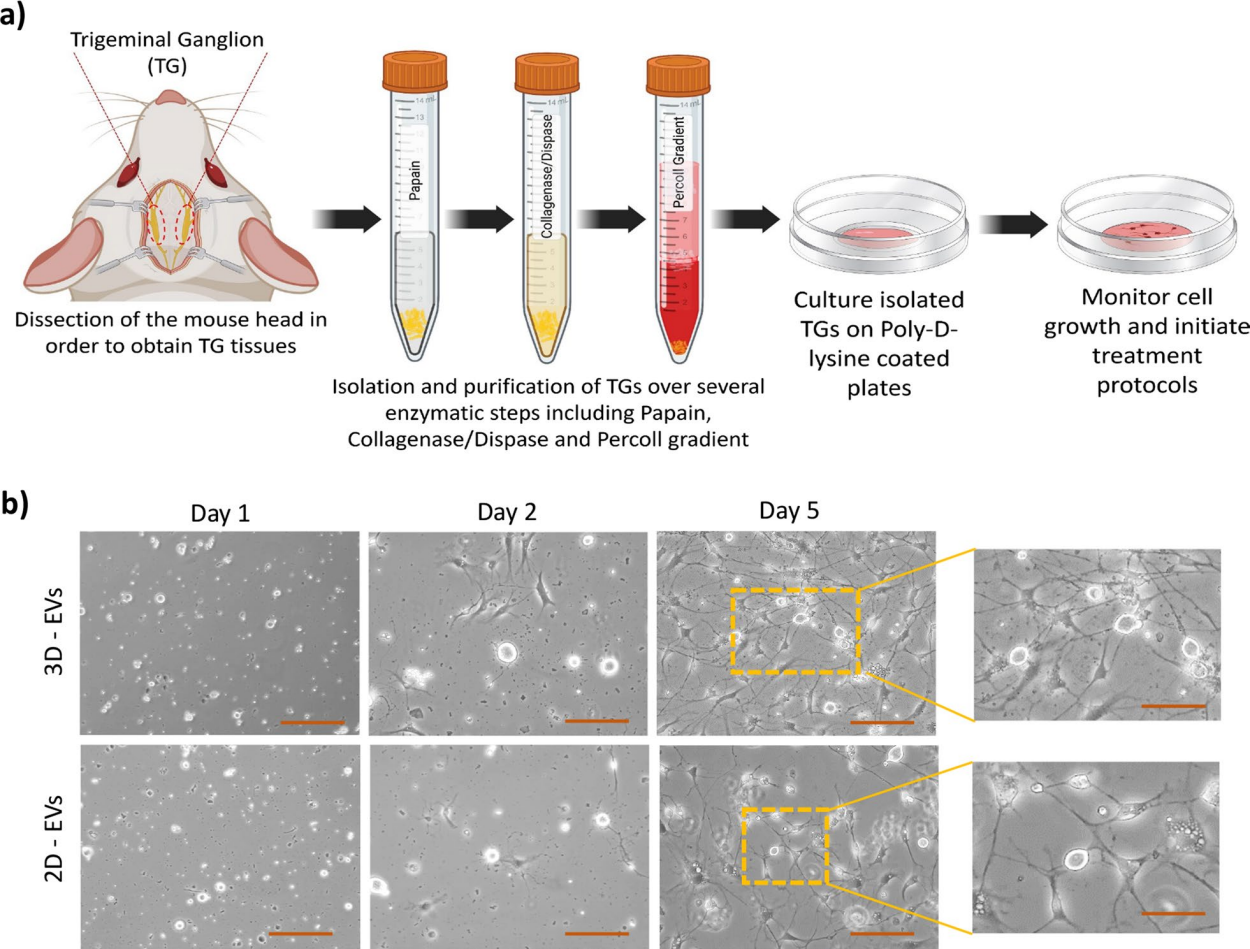
Trigeminal ganglion (TGs) cells isolation and elongation in the presence of EV treatment. **a** Schematic diagram of experimental procedure for TG cells isolation from BALB/c mice by utilizing different enzymatic procedures and plating the cells in PDL-coated dishes. **b** Representative images taken using bright-field microscopy from dishes treated with either 3D- or 2D-EVs over 5 days showed considerable neurite growth and elongation in both conditions with a remarkable increase in the number of neurite sprouts in 3D-EVs condition versus 2D-EVs. Scale bars in D1 to D5 images are 200px, and in inlets, they are 100px

To assess the effect of BM-MSC-derived EVs on neurite growth and elongation in vitro, TGs were isolated from 3–5-week-old BALB/c mice and plated on PDL-coated dishes (Fig. 4a). After 24 h, TGs were treated either with 40 × 10^11^ EVs/mL resuspended in collection media or with EV-depleted media as a control, which is the collection media supernatant obtained after ultracentrifugation.

Morphological observation using a bright-field microscope illustrated that at the time of plating, the TGs appeared spherical with no apparent neurite outgrowth. Treatments were applied 24 h post-plating when thin neurites began to emerge from the cell bodies. Neurite growth and elongation were monitored over 5 days. We found that the EVs treatments promoted neurites length and branching in both 3D-EVs and 2D-EVs conditions. Additionally, neurite elongation and branching were notably higher in 3D-EVs than in 2D-EVs treatments. The neurites growth and the number of sprouts that were observed to emerge from the cell bodies were considerably higher in 3D-derived EVs condition compared to 2D-derived EVs, which was indicative of neuronal growth with more complexity (Fig. 4b). Treatment with a medium depleted of EVs (control groups) showed no effect on neurites elongation and branching (Additional file 1: Figure S4).

### 3D-derived EVs increases neurite elongation and complexity

**Fig. 5:**
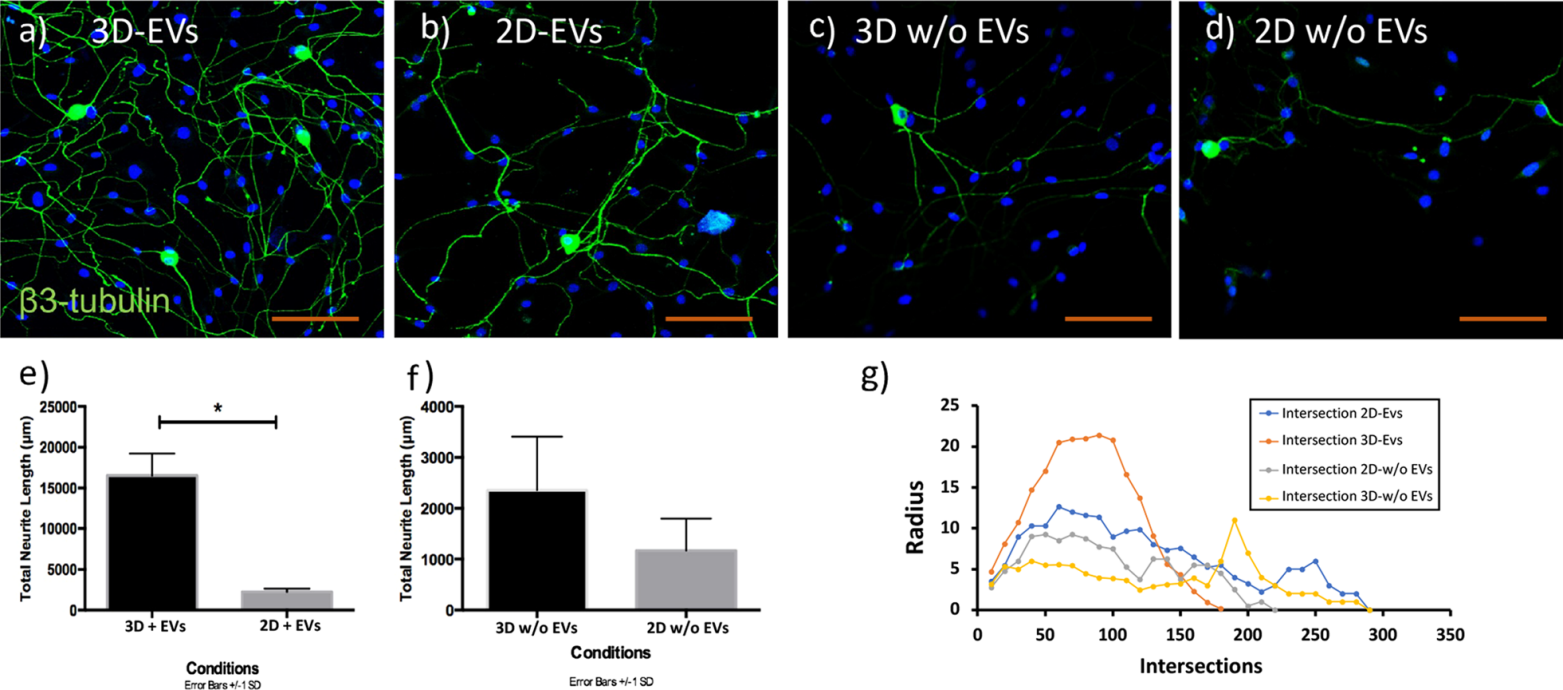
3D-EVs induce greater neurite elongation and branching than 2D-EVs. **a**, **b** Representative confocal microscopy images of TG neurite elongation cultured under 3D-EVs and 2D-EVs. **c**, **d** Neurite growth in EV-depleted media derived from 3D or 2D cultures. **e** Quantification of total neurite growth demonstrated that 3D-EVs induce significant growth than 2D-EVs. **f** No length difference was observed in cultures treated with EV-depleted media. Statistical significance was tested with an unpaired student t test (two-tailed) for evaluating differences between the two groups, and statistical significance was determined (*P* < 0.05) (*n* = 4 biological replicate & *n* = 16 technical replicates for each condition). **g** Sholl analysis performed in the dendritic tree demonstrated that 3D-EVs significantly increased the number of dendritic intersections between 50 and 120 radii compared to 2D-EVs and control conditions. Data represent the mean ± 1 SD of 3 independent experiments. *P* < 0.05. Scale bar 100 µm

The analysis of neurite growth and length was performed in β3 tubulin immunostained TG neurons. Neurite length was quantified using Neurolucida software, and the average neurite size was compared between two conditions. The immunostaining data showed that 3D-derived EVs induced significant elongation in TG neurons compared to 2D-derived EVs after 5 days (3301.5 μm vs. 1860.5 μm, *P* < 0.05) (Fig. 5a and b). Also, Sholl analysis performed to study the complexity of neurons indicated that 3D-derived EVs induced a sevenfold higher neurite elongation than 2D-derived EVs.

The impact of 3D-EVs on total neurite growth with an elongation length of 14,588.2 μm was statistically significant than the effect of 2D-EVs with an elongation length of 2243.96 μm (Fig. 5e, *P* < 0.05). No significant difference was observed between the 3D Depleted-EVs versus 2D Depleted-EVs (Fig. 5f). Furthermore, each EV condition (+EV) was compared with its negative control (−EVs), and the results demonstrated a significant increase in total neurite length in +EV versus −EV condition in both 3D and 2D conditions (*P* < 0.05), (Additional file 1: Figure S5). Additionally, 3D-EVs significantly increased neurite branching, and complexity compared to other conditions (Fig. 5f, *P* < 0.05).

## Discussion

Extracellular vesicles are believed to be essential for cellular communication as they transfer functional proteins, metabolites, and nucleic acids to recipient cells [19, 52–54]. Many studies have shown the beneficial effect of EVs in preclinical applications. However, the lack of a platform to obtain sufficient and consistent MSC-EVs remains a challenge for therapeutic development. It is worth mentioning that there are several terminologies used for extracellular vesicles in different studies (e.g., exosomes or microsomes). Since EVs were not isolated based on size in current study, term EVs have been used throughout the manuscript.

The aim of this study was to investigate the effect of BM-MSCs grown in 2D monolayer cultures and 3D bioreactor systems on EV production, their molecular characteristics, and regenerative potentials. Followed by successful expansion of BM-MSCs in both 2D and 3D culture systems, EVs were collected and were characterized with NanoSight NS300 for particle size and concentration. Although the results revealed a remarkable increase (24-fold change) of EVs concentration obtained from 3D culture systems compared to conventional 2D cultures, the difference was found to be statistically insignificant probably due to the low number of repeats (*n* = 4). Results obtained from the DLS analysis of 2D cultures showed a bimodal distribution of EVs with a large exosome population defined at 60–180 nm and a smaller peak for larger EVs (300–400 nm). However, for 3D-derived EVs, DLS analysis demonstrated multimodal distribution, with detected particles mostly between 50 and 170 nm and less abundant EVs defined at 200–300 nm and 400 nm.

It is well known that the NTA size distributions suffer from the inability to accurately measure the smaller constituents in a heterogeneous population. Small EVs are obscured by larger EVs which influences both size and concentration measurements detrimentally [55]. Therefore, another measurement for EVs with ExoView was further performed to produce more accurate size distributions and to provide the ability to discriminate size distributions of specific subpopulations of EVs expressing specific protein biomarkers [56]. Consistent with the NTA results, ExoView analysis demonstrated a substantial increase in EV concentration in 3D versus 2D conditions. Furthermore, ExoView analysis illustrated the expression of three tetraspanin positive particles, CD63, CD81, and CD9. The acquired results were consistent with our previous study which revealed the expression of CD63, CD81, and CD9 markers in exosomes isolated from preserved cornea samples [57]. Moreover, the data emphasized that EVs obtained from 3D cultures possess a higher copy number of tetraspanins per EV compared to vesicles from 2D cultures. Additionally, colocalization analysis further highlighted the more heterogeneous tetraspanin phenotype associated with 3D culture conditions. Results obtained in this study were consistent with previous studies demonstrating higher yield in extracted EVs from 3D cultures with significantly different expression profiles compared to conventional 2D cultures [47, 58].

The in vitro effects of both 2D and 3D-derived EVs with respect to their neuro-regeneration potential were examined next. Corneal nerves are supplied by the upper most ophthalmic branch of trigeminal nerves in the brain [59]. Thus, in in vitro studies, extraction of trigeminal cells from brain are more feasible than isolating the corneal nerves directly and have been used in our paper and also others [60]. In our study, we showed that both 2D and 3D-derived EVs enhance neurite growth and elongation. However, 3D-derived EVs showed significantly enhanced axonal growth, elongation, and complexity. 3D cell culture allows MSCs to enhance growth and increase extracellular matrix interactions which, in turn, influences their paracrine signaling activity. This could subsequently affect the EV biogenesis and secretion, and therefore, impact the neuro-regeneration properties of MSC-derived EVs [61].

The beneficial effects exerted by MSC-EVs in different CNS and peripheral nerve injuries have been highlighted in different studies [62–65]. It has been established that MSC-EVs are enriched with multiple neurotrophic factors which provide the possibility of regulating neuronal survival and recovery after injury and axonal outgrowth [62]. It is demonstrated that adipose-derived MSC-exosomes could be internalized by Schwan cells (SC) and axons in vitro and in vivo which could significantly increase the proliferation of SC and promote axonal regeneration [66]. Also, exosomes derived from human-induced pluripotent stem cell-derived neural progenitor cells were shown to protect neuronal function under ischemic conditions [67]. It is noteworthy to mention that stem cell transplantation solely can be therapeutically beneficial, but these grafted cells do not directly replace lost tissues; they rather act in more indirect manners, in which the secretion of EVs appears to be a critical factor [68]. As opposed to cells, EVs have no nucleus and cannot self-replicate; therefore, they do not possess any intrinsic tumorigenic properties. Furthermore, the small size of EVs facilitates filter sterilization of them and compared to stem cells, handling, and storage of EVs is significantly easier. EV-based therapeutics, therefore, offer several advantages over conventional direct cellular approaches, resulting in an increasing number of translational studies involving MSC-EVs [69].

Recently, the use of EVs in ocular diseases has been attracting attention as well. In a study conducted in our group, it was shown that EVs derived from human corneal MSCs could accelerate corneal epithelial wound healing [57]. Other studies also demonstrated that EVs derived from normal human corneal limbal keratocytes can enhance proliferation and wound healing rates of primary limbal epithelial cells, likely via activating Akt signaling [70]. Bai et al. assessed local administration of umbilical cord MSC-exosomes on mice with autoimmune uveitis. Reduced intensity of autoimmune uveitis with reduced infiltration of T cells, as well as reduced effects of CCL2 and CCL21, chemo-attractive factors for inflammatory cells, were established in their studies [37]. Exosomes derived from human adipose tissue have been applied in the form of eye drops to mice with damaged ocular tissue from dry eye diseases and were found to reduce inflammasome formation and IL-1B signaling, suppressing the inflammatory response and alleviating ocular surface damage [71]. Furthermore, BM-MSC-derived EVs have been injected intravitreally into rat models of glaucoma and shown significant neuroprotection effect in retinal ganglion cells, while preventing degenerative thinning and atrophy [72].

Future studies need to investigate whether enhanced biological functions of EVs are only due to the concentrations of EVs, or increased number of epitopes per EVs would alter their efficacy, or if the therapeutically efficacious component of the isolated EVs may indeed be an EV subtype that can be further purified. The number and type of EVs could be potentially important in multifactorial diseases where multitargets effects could be better suited for repair, such as in corneal wound healing where nerve and epithelium regeneration are essential to recover function. Also, extended studies are needed to refine the mechanisms of action of these EVs. The therapeutic effect of EVs can be enhanced by engineering measures to reduce necessary doses and administrations when utilized. For instance, exposing MSCs to the inflammatory cytokines as an engineering measure can enhance the neuroprotection effect of derivative EVs [73].

## Conclusion

In this study, our data demonstrated that the concentration and heterogeneity of MSC-derived EVs are highly impacted by culture conditions. BM-MSCs-derived EVs can promote neurite elongation and growth. Additionally, EVs derived from 3D-cultured BM-MSCs demonstrated more potent biological functions than those derived from cells grown in conventional 2D flask cultures. The outcomes of this study highlight critical differences in EVs obtained from different culture microenvironments, which should be considered when scaling up MSC cultures for clinical manufacturing of EVs. Our findings also suggest that human BM-MSCs-derived EVs may represent a novel therapeutic approach to the management of corneal nerve injury.

## Supplementary Information

**Additional file 1. Supplementary Figure 1.** Comparison between 3D and 2D-derived EVs. Representative videos from 3D-derived EVs and 2D-derived EVs from NanoSight. Also, statistical analysis demonstrated that 3D-dervied EVs had considerably higher number of EVs compared to 2D but this difference was not statistically significant.

## Data Availability

Not applicable.

## Abbreviations

MSC: Mesenchymal stem cell
BM: Bone marrow
BM-MSCs: Bone marrow mesenchymal stem cells
EVs: Extracellular vesicles
PDL: Poly-d-lysine
NTA: Nanoparticle tracking analysis
TGs: Trigeminal cells
CNS: Central nervous system
