# Supplementary material for: Bone marrow mesenchymal stromal cells in a 3D system produce higher concentration of extracellular vesicles (EVs) with increased complexity and enhanced neuronal growth properties
Source: Stem Cell Res Ther. 2022 Aug 19;13:425. doi: 10.1186/s13287-022-03128-z (PMC9389821; doi:10.1186/s13287-022-03128-z)
Supplement: Supplementary file 1 — Additional file 1. Supplementary Figure 1. Comparison between 3D and 2D-derived EVs. Representative videos from 3D-derived EVs and 2D-derived EVs from NanoSight. Also, statistical analysis demonstrated that 3D-dervied EVs had considerably higher number of EVs compared to 2D but this difference was not statistically significant. [file 13287_2022_3128_MOESM1_ESM.docx]

**Supplementary data**

**Bone marrow mesenchymal stem cells cultured in a 3D-sytem produced higher concentration of extracellular vesicles (EVs) with increased complexity and enhanced neuronal growth properties**

Elmira Jalilian^1, 2*^, Hamed Massoumi^1, 2^, Bianca Bigit^1^, Sohil Amin^1^, Eitan A. Katz^1^, Victor H. Guaiquil^1^, Khandaker N. Anwar^1^, Peiman Hematti, MD^3^, Mark I. Rosenblatt^1^, Ali R. Djalilian^1*^

^1^ Department of Ophthalmology and Visual Sciences, Illinois Eye and Ear Infirmary. University of Illinois at Chicago, Illinois, United States.

^2^ Richard and Loan Hill Department of Bioengineering, University of Illinois at Chicago, Chicago, IL, United States.

^3^ Department of Medicine, Hematology/Oncology Division, University of Wisconsin-Madison, School of Medicine and Public Health, Madison, WI 53705, USA

**Supplementary data S1.**


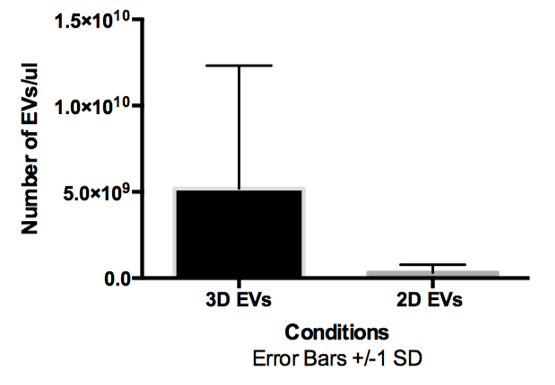

*Supplementary Figure 1*. Comparison between 3D and 2D-derived EVs. A video demonstrating an example of the dynamic light scattering method for each of the 2D-EV and 3D-EV samples using Nanosight.

**Supplementary data S2.**


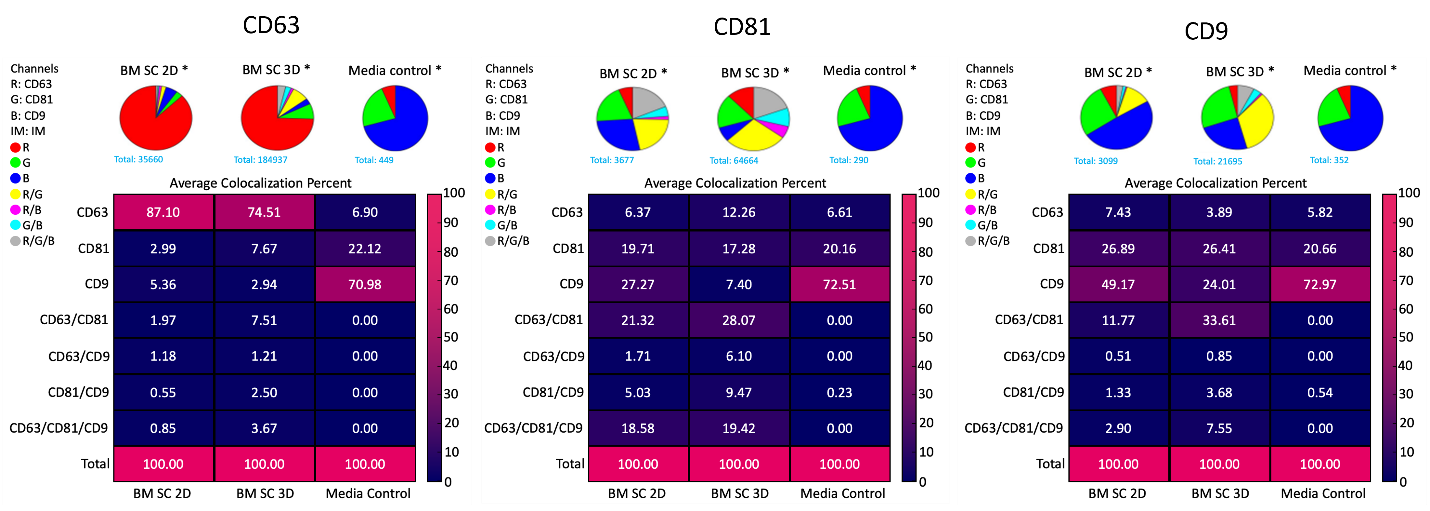


Supplementary Figure 2. Co-localization percent of all three markers. Average of co-localization percent of all three markers CD81, CD63 & CD9. Fluorescence data can be analyzed to determine the degree of colocalization of different markers.

**Supplementary data S3.**


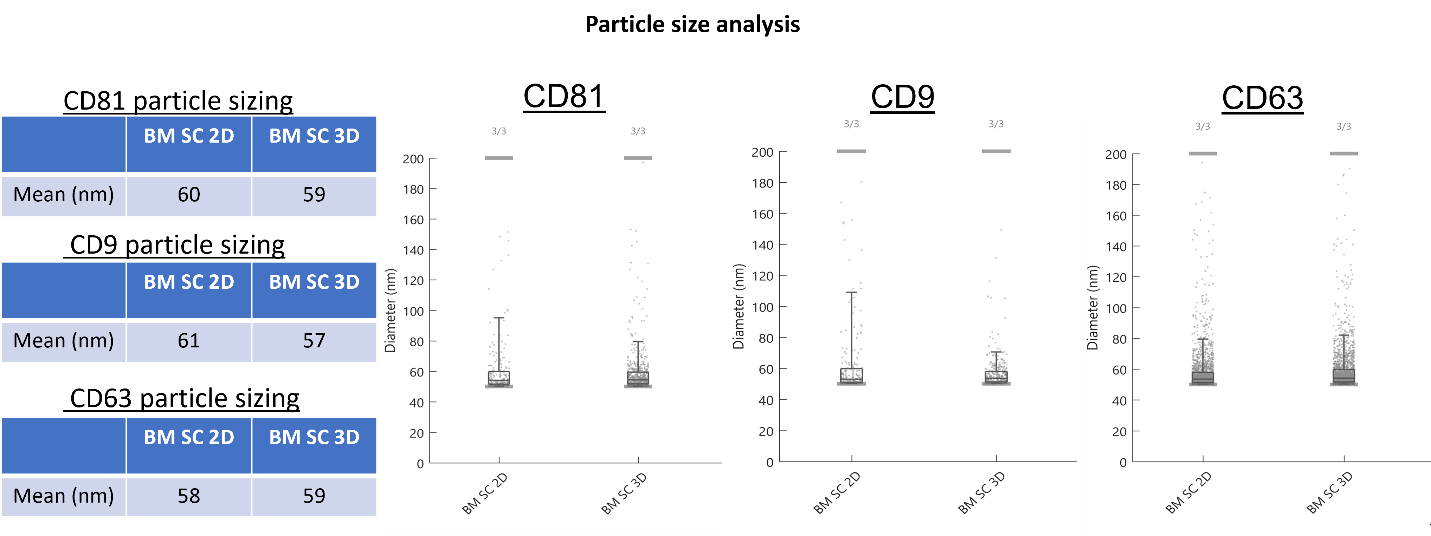


*Supplementary Figure 4*. Particle size analysis.Particles between 50 – 200nm in diameter are sized. Sizing is obtained by interferometry-based label free measurements performed on each spot. The mean is calculated from three spots for each capture antibody.

**Supplementary data S4.**


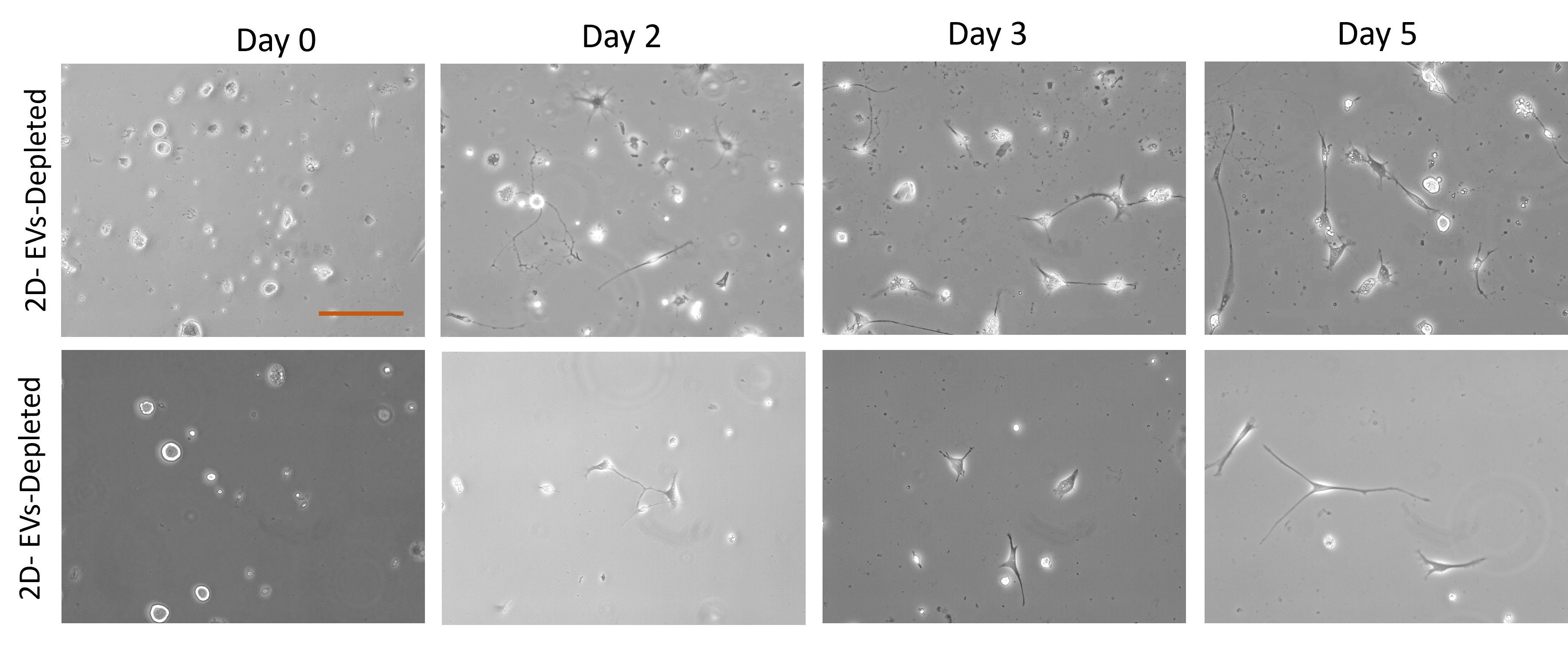


*Supplementary Figure 4*. Treatment with a medium depleted of EVs showed no effect on neurites elongation and branching. Scale bar 200px.

**Supplementary data S5.**


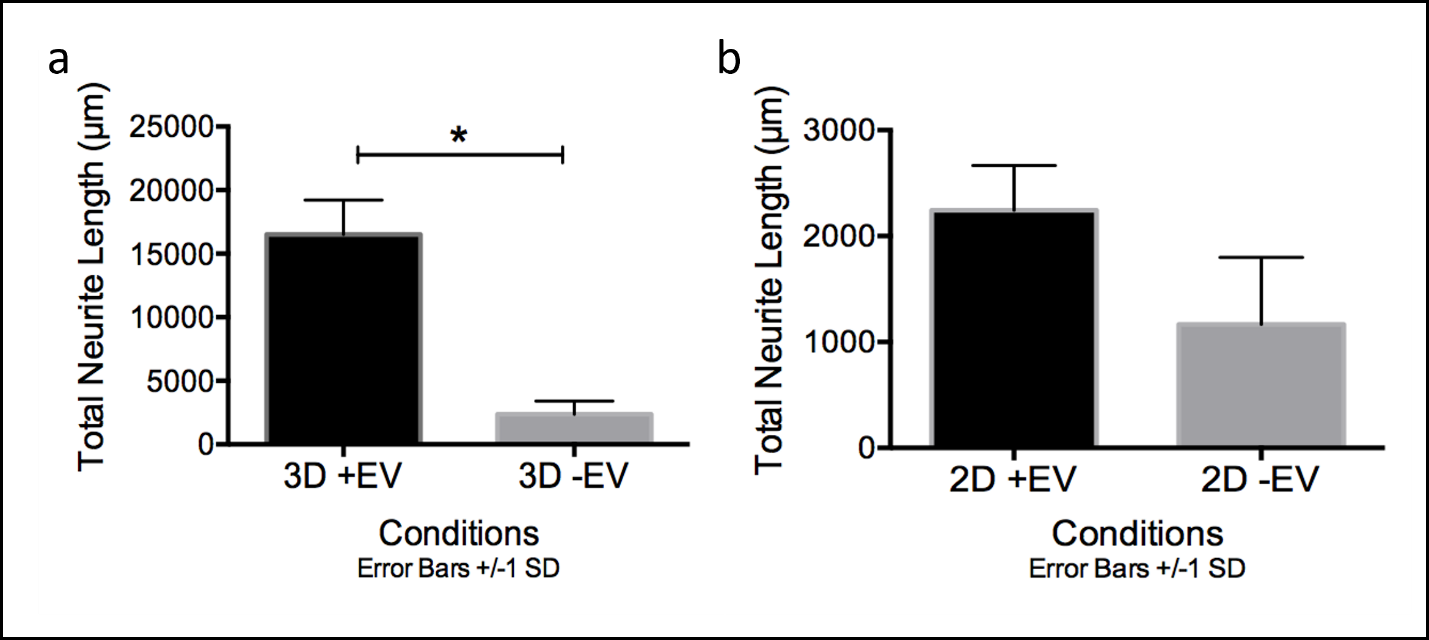


*Supplementary Figure 5.* Quantification of total neurite growth demonstrated that 3D-EVs induces significant growth than 3D-w/o EVs. (f) No length difference was observed in cultures treated with EV-depleted media. Statistical significance was tested with an unpaired student t-test (two tailed) for evaluating differences between two groups and statistical significance was determined (P<0.05)
